# Retraction: Improvement of non-invasive tests of liver steatosis and fibrosis as indicators for non-alcoholic fatty liver disease in type 2 diabetes mellitus patients with elevated cardiovascular risk profile using the PPAR-α/γ agonist aleglitazar

**DOI:** 10.1371/journal.pone.0339230

**Published:** 2025-12-19

**Authors:** The PLOS One Editors

After this article [1] was published, the corresponding author contacted the journal to raise concerns about the validity of the results in [1] and request retraction of the article. Specifically, they stated that whilst conducting further analysis on the AleCardio trial dataset underlying this article [1], they identified the following errors in variable labels:

- The variable serum glutamic-oxaloacetic transaminase (SGOT) was incorrectly renamed as alanine aminotransferase (ALT). The correct alternative terminology for this enzyme is aspartate aminotransferase (AST).
- The variable serum glutamic-pyruvic transaminase (SGPT) was incorrectly renamed as AST. The correct alternative terminology for this enzyme is ALT.

The corresponding author stated that this error affects 3 of the 4 outcomes evaluated in [1] including liver fat score (LFS), NAFLD fibrosis score (NFS), and fibrosis 4 score (FIB-4), which are calculated using both AST and ALT measurements. They further stated that when the corrected dataset is analyzed, the results for markers of fibrosis (FIB-4 and NFS) are reversed and suggest a negative effect following treatment with aleglitazar, and not an improvement, as claimed in this article [1]; the results and conclusions based on the corrected analysis of markers of steatosis (LFS and Liver Accumulation Product) remain similar to those reported in [1].

In light of the above error in the handling of the underlying dataset, which means the key conclusion relating to improved fibrosis outcomes following aleglitazar treatment is no longer supported by the reported results, and at the request of the authors, the *PLOS One* Editors retract this article.

All authors agreed with the retraction.
